# Risk Factors and Clinical Outcomes in Preterm Infants with Pulmonary Hypertension

**DOI:** 10.1371/journal.pone.0163904

**Published:** 2016-10-07

**Authors:** Joseph M. Collaco, Gul H. Dadlani, Melanie K. Nies, Jenny Leshko, Allen D. Everett, Sharon A. McGrath-Morrow

**Affiliations:** 1 Eudowood Division of Pediatric Respiratory Sciences, Johns Hopkins Medical Institutions, Baltimore, Maryland, United States of America; 2 Division of Pediatric Cardiology, Nemours Children’s Hospital, Orlando, Florida, United States of America; 3 The Helen B. Taussig Congenital Heart Center, Johns Hopkins Medical Institutions, Baltimore, Maryland, United States of America; 4 Division of Pediatric Cardiology, All Children’s Hospital, Johns Hopkins Medicine, St. Petersburg, Florida, United States of America; Centre Hospitalier Universitaire Vaudois, FRANCE

## Abstract

**Background:**

Pulmonary hypertension (PH) is a significant cause of morbidity in preterm infants, but no screening guidelines exist. We sought to identify risk factors and clinical outcomes associated with PH in preterm infants to develop a PH risk score.

**Methods:**

Retrospective analysis of two separate populations of preterm infants (NICU cohort n = 230; Clinic registry n = 580).

**Results:**

8.3% of the NICU cohort had PH after 4 weeks of age, while 14.8% of the clinic registry had PH after 2 months of age. Lower birth weights and longer initial hospitalizations were associated with PH in both populations (*p*<0.001 for all tests). Using adjusted logistic regression, patent ductus arteriosus (PDA) requiring ligation was associated with PH in both the NICU cohort (OR: 3.19; *p* = 0.024) and the clinic registry (OR: 2.67; *p*<0.001). Risk factors (birth weight ≤780 grams, home supplemental oxygen use, and PDA ligation) identified in the clinic registry (training dataset) were validated in the NICU cohort with 0–1 factors present were associated with ≤1.5% probability of having PH, any 2 factors with a 25% probability, and all 3 factors with a 40% probability.

**Conclusions:**

Lower birth weight, PDA ligation, and respiratory support were associated with PH in both populations. A PH risk score based on clinical indicators from the training dataset predicted PH in the validation set. This risk score could help focus resources to preterm infants at higher risk for PH. Further work is needed to determine whether earlier or more aggressive management of ductal lesions could alter PH outcomes.

## Introduction

Of the approximately 4 million live births annually in the United States, 9.6% of these births in 2014 were premature (less than 37 weeks gestation) with 1.4% born weighing less than 1500 grams.[1] A common complication of premature birth is bronchopulmonary dysplasia (BPD), a chronic lung disease of infancy characterized by features such as supplemental oxygen dependency, wheezing, tachypnea, dyspnea, etc.[2] The rates of BPD vary by center, but may affect at least 25% of those weighing less than 1500 grams at birth,[3] or at least 14,000 infants in the United States annually.

An emerging co-morbidity of BPD is pulmonary hypertension (PH), which may affect 14–43% of preterm infants with BPD in prospective and retrospective studies.[4–9] This co-morbidity in the BPD population is associated with increased morbidity and mortality when compared to infants with BPD without PH.[10] PH is associated with a mortality rate ranging from 14% to 38% in these same studies.[4–9] Infants with PH and BPD have been shown to spend an additional 2.2 months in the hospital compared to infants with BPD alone, resulting in at least an additional $198,000 in health care costs.[11] Thus, it is critical to identify the risk factors that may lead to the development of PH. The development of PH is complex, likely arising from an interplay of genetic and pre-/post-natal environmental factors.[12] These may include alterations in angiogenic pathways,[13–16] possible genetic modifiers,[17,18] intrauterine growth,[5,8,10,19,20] oligohydramnios,[6,10] patent ductus arteriosus (PDA),[4] and placental vascularity.[21,22]

Results from echocardiography have been used to identify infants with PH and several recent studies have demonstrated the utility of echocardiograms in identifying preterm infants at high risk for BPD and PH.[8,9,23] Thus, in this study we attempted to identify a subpopulation of preterm infants at highest risk for developing PH to focus the deployment of echocardiographic resources most efficiently. In addition to understanding risk factors for the development of PH, it is also important to recognize other co-morbidities and outcomes that may be associated with it in order to pre-emptively counsel families and be pre-cognizant of potential complications. Although severe BPD has been linked to PH,[9,19,23] the potential association of PH with the frequency of other common co-morbidities of preterm birth is poorly described.

In this study we sought to identify risk factors and outcomes associated with pulmonary hypertension in preterm infants using two existing databases of preterm infants, and to identify a potential predictive score for PH. The first population is a retrospectively recruited cohort of preterm infants recruited from a BPD subspecialty clinic (to be used as a training dataset).[11] The second (non-overlapping) population is a smaller prospectively recruited cohort from an NICU at a tertiary care children’s hospital (to be used as a validation dataset).[24] Although retrospective, the first population has the advantage of being enriched for the diagnosis of PH on the basis of having BPD.

## Methods

### BPD Clinic Study Population

Subjects (n = 580) in this population were recruited from the outpatient Johns Hopkins Bronchopulmonary Dysplasia Clinic (Baltimore, MD) between January 2008 and September 2015. Local neonatologists and pediatricians refer preterm infants with respiratory disease to the clinic for follow up care, which is staffed by two board-certified pediatric pulmonologists. Inclusion criteria are being born preterm (≤36 weeks gestation) and being diagnosed with BPD by a pediatric pulmonologist or neonatologist per NICHD criteria.[25] Infants were excluded if they had hemodynamically significant congenital heart disease. This study was approved by the Johns Hopkins University Institutional Review Board (NA_00051884), and verbal informed consent was obtained from parents/guardians; this expedited consent was approved by the IRB owing to the minimal potential risks incurred by the participants.

### NICU Study Population

Subjects (n = 230) in this population were recruited from the All Children’s Hospital NICU (St. Petersburg, FL) between January 2008 and December 2011. Inclusion criteria were any admitted infant born ≤1000 grams. Infants were excluded if they had hemodynamically significant congenital heart disease or they did not survive >30 days of life; patent ductus arteriosus alone was not an exclusion criterion. This study was approved by the All Children’s Hospital Institutional Review Board (IRIS 12–0500), and written informed consent was obtained from parents/guardians.

### Pulmonary Hypertension

Subjects were categorized as having PH based on clinical echocardiograms after 4 weeks of age for the NICU population and 2 months of age for the BPD clinic population.[11,24] Although the diagnostic gold standard for PH remains cardiac catheterization with a mean pulmonary artery pressure ≥25 mmHg, in this population of preterm infants with frequently multiple medical comorbidities, echocardiography was used for noninvasive evaluation of pulmonary pressures. Using the modified Bernoulli equation (TRJV^2^ × 4), systolic pulmonary artery pressure was estimated with an assumed right atrial pressure of 5 mmHg. PH was diagnosed by elevated right ventricular pressure as estimated by tricuspid regurgitation jet or PDA gradient. If neither of those measures were available or adequate, a flattened systolic interventricular septal position was used to qualitatively describe RV pressure as greater than ½ systemic pressure. All echocardiograms were reviewed by a board-certified pediatric cardiologist.

### Demographics/Clinical Data

As existing databases were used for this study, not all data elements were available for both populations. Type of insurance was established using billing records. Median household income was derived from 2010 U.S. census tract data using residential zip codes (U.S. median household income: $50,502; State of Maryland median household income: $70,004). Birth weight percentiles reflect birth weights corrected for gestational age.[26] Race/ethnicity and primary caregiver education level were self-reported. The presence of gastrostomy tubes, tracheostomies, Nissen fundoplication, cerebrospinal fluid shunt, PDA requiring ligation, home oxygen and/or ventilator use, sepsis evaluation, and specific medication use as well as initial discharge date and date of first BPD clinic visit were ascertained through chart review. Sepsis evaluation was defined as the initiation of a work-up for sepsis, and not necessarily an episode of proven sepsis. PDA ligation was pursued at the clinical discretion of the attending neonatologist in both populations.

### Statistical Methods

Chi-square and *t* tests were used to compare demographic and clinical features. Statistically significant variables from chi-square and *t* tests were used to build adjusted multivariate regression models. STATA IC 11 (StataCorp LP, College Station, TX) was used for analyses. *P* values <0.05 were considered statistically significant.

## Results

### Demographics

Among the 230 subjects in the NICU population, 19 (8.3%) had pulmonary hypertension after 4 weeks of age (**Table 1**), and among the 580 subjects in the BPD outpatient clinic population, 86 (14.8%) had pulmonary hypertension after 2 months of age (**Table 2**). Of the factors of gestational age, birth weight, and birth weight percentile, only lower birth weights were associated with PH in both populations (*p*<0.001 in both populations). No proxy measurements for socioeconomic status (insurance status: *p* = 0.97, estimated household income: *p* = 0.19, or caregiver educational level: *p* = 0.25) were associated with PH in the BPD clinic population. In the NICU population, the length of stay was 1.7 months longer in those with PH (*p*<0.001) compared to those who did not have PH, and likewise in the BPD clinic population, infants with PH spent an additional 2.9 months in the hospital prior to initial discharge compared to infants without PH (*p*<0.001). These differences in discharge age with the presence of PH persisted even after adjustment for birth weight using regression (1.3 months longer in the NICU population, *p*<0.001, n = 229; 2.6 months longer in the BPD clinic population, *p*<0.001, n = 563). Similarly, subjects in the BPD clinic population with PH presented to BPD clinic 1.5 months later than those who did not have PH (*p* = 0.027). There were no differences in the frequency of PH in the 2 populations by sex, and no difference in PH by race/ethnicity in the BPD clinic population. Perhaps not unexpectedly, owing to different recruitment criteria, the two study populations differed by sex frequency, gestational age, birth weight, age at discharge, frequency of BPD, home supplemental oxygen use, and presence of PH, but not birth weight percentile, frequency of PDA ligation, and presence of tracheostomy (**S1 Table**).

**Table 1:** NICU Population Demographics and Clinical Features.

Mean ± S.D.[Range]	Entire NICU Population	Pulmonary Hypertension after 4 weeks of age	No Pulmonary Hypertension after 4 weeks of age	*P* Value
N (unless otherwise specified)	230	19	211	
**DEMOGRAPHICS**				
Sex (% female)	50.9%	52.6%	50.7%	0.87
Gestational Age (weeks)	25.9 ± 1.6 [22.0, 29.0]	25.7 ± 2.0 [23.0, 29.0]	25.9 ± 1.5 [22.0, 29.0]	0.62
Birth Weight (grams)	788 ± 145 [330, 1000]	667 ± 144 [425, 930]	799 ± 140 [330, 1000]	<0.001
Birth Weight Percentile (%)	41 ± 21 [1, 91]	30 ± 28 [2, 84]	42 ± 20 [1, 91]	0.019
**CLINICAL FEATURES**				
Patent Ductus Arteriosus Requiring Ligation (% yes)	22.7% (n = 229)	52.6%	20.0% (n = 210)	0.001
Bronchopulmonary Dysplasia (% yes)	67.4%	89.5%	65.4%	0.032
Home Supplemental Oxygen (% yes)	23.1% (n = 229)	84.2%	17.6% (n = 210)	<0.001
Tracheostomy (% yes)	3.5%	10.5%	2.8%	0.08
Intraventricular Hemorrhage (% yes)	25.8% (n = 229)	36.8%	24.8% (n = 210)	0.25
Necrotizing Enterocolitis (% yes)	14.4%	21.1%	13.7%	0.38
Sepsis Evaluation (number of episodes)	4.4 ± 3.3 [0, 21] (n = 229)	8.0 ± 4.1 [3, 16]	4.0 ± 3.0 [0, 21] (n = 210)	<0.001
Retinopathy of Prematurity (% yes)	17.0%	31.6%	15.6%	0.08

**Table 2:** BPD Clinic Population Demographics and Clinical Features.

Mean ± S.D.[Range]	Entire BPD Clinic Population	Pulmonary Hypertension after 2 months of age	No Pulmonary Hypertension after 2 months of age	*P* Value
N (unless otherwise specified)	580	86	494	
**DEMOGRAPHICS**				
Sex (% female)	39.1%	45.3%	38.1%	0.20
Race/Ethnicity (% non-white)	63.8%	66.3%	63.4%	0.60
Gestational Age (weeks)	27.1 ± 2.9[22.7, 36.9]	25.9 ± 2.5[23.0, 36.7]	27.3 ± 3.0[22.7, 36.9]	<0.001
Birth Weight (grams)	994 ± 511[380, 4063](n = 565)	821 ± 468[380, 3370]	1025 ± 512[380, 4063](n = 479)	<0.001
Birth Weight Percentile (%)	41 ± 23[1, 96](n = 565)	39 ± 25[1, 96]	41 ± 23[1, 95](n = 479)	0.40
Discharge Age (months)	4.2 ± 2.9[0.1, 24.5] (n = 577)	6.6 ± 4.1[0.4, 24.5]	3.7 ± 2.4 [0.1, 24.4] (n = 491)	<0.001
First Pulmonary Clinic Appointment Age (months)	7.7 ± 6.0 [0.8, 51.3]	9.0 ± 5.0 [2.7, 33.7]	7.5 ± 6.1 [0.8, 51.3]	0.027
Public Insurance (% yes)	57.9	58.1	57.9	0.97
Estimated Household Income ($ 000’s)	64.0 ± 21.9 [15.6, 156.6]	66.9 ± 23.8 [15.6, 132.7]	63.5 ± 21.6 [15.6, 156.6]	0.19
Caregiver Education (%): Less than High School, High School Graduate, Some College, College Graduate, Any Graduate Education	7.6%, 22.4%, 30.0%, 24.1%, 16.0% (n = 370)	11.5%, 23.0%, 24.6%, 31.2%, 9.8% (n = 61)	6.8%, 22.3%, 31.1%, 22.7%, 17.2% (n = 309)	0.25
**CLINICAL FEATURES**				
Patent Ductus Arteriosus Requiring Ligation (% yes)	19.6%(n = 576)	39.5%	16.1% (n = 490)	<0.001
Home Supplemental Oxygen (% yes)	35.7%	69.8%	29.8%	<0.001
Tracheostomy (% yes)	4.3%	10.5%	3.2%	0.002
Home Mechanical Ventilation (% yes)	3.3%	10.5%	2.0%	<0.001
Inhaled Corticosteroids (% yes)	80.7%	91.9%	78.7%	0.004
Diuretics (% yes)	59.0% (n = 576)	83.7%	54.7% (n = 490)	<0.001
Cerebrospinal Fluid Shunt (% yes)	8.6%	12.8%	7.9%	0.14
Gastrostomy Tube (% yes)	26.2%	60.5%	20.2%	<0.001
Nissen (% yes)	17.1%	38.4%	13.4%	<0.001

### Respiratory Clinical Features

Almost every measure of respiratory disease in both populations was associated with pulmonary hypertension, including home supplemental oxygen use (*p*<0.001 in both populations), home ventilator use (*p*<0.001 in the BPD clinic population), having a tracheostomy (*p* = 0.08 in the NICU population; *p* = 0.002 in the BPD clinic population), the use of diuretics (*p*<0.001 in the BPD clinic population), and the use of inhaled corticosteroids (*p* = 0.004 in the BPD clinic population)(**Tables 1 and 2**).

Multivariable logistic regression was used to test which outcomes were still associated with PH after adjustment for the potential confounder of birth weight as PH was associated with lower birth weights, as are many morbidities of prematurity. Assessing all significant respiratory clinical features simultaneously with adjustment for birth weight, home supplemental oxygen (OR: 4.07; 95%CI: 2.42, 6.86; *p*<0.001), home mechanical ventilation (OR: 4.33; 95%CI: 1.51, 12.44; *p* = 0.007), and diuretic use (OR: 2.76; 95%CI: 1.47, 5.17; *p* = 0.002) continued to be associated with PH in the BPD clinic population (n = 562). However, the respiratory clinical features of the NICU population (n = 230), specifically home supplemental oxygen (*p* = 0.78) or bronchopulmonary dysplasia (*p* = 0.31) were not associated with PH in adjusted regression.

### Cardiovascular Clinical Features

In both populations, pulmonary hypertension was associated with PDA ligation in those with PH (*p* = 0.001 in the NICU population; *p*<0.001 in the BPD clinic population). Again using multivariable logistic regression adjusted for birth weight, PDA requiring ligation was associated with PH in both the NICU population (OR: 3.19; 95%CI: 1.16, 8.77; *p* = 0.024; n = 229) and the BPD clinic population (OR: 2.67; 95%CI: 1.60, 4.46; *p*<0.001; n = 562). Among the 86 subjects with PH in the BPD clinic population, 20.9% were on sildenafil and 4.7% were on sildenafil and bosentan, and 74.4% were not on any pulmonary anti-hypertensive agents.

### Neurological Clinical Features

Pulmonary hypertension was not associated with intraventricular hemorrhage in the NICU population (*p* = 0.25) nor placement of cerebrospinal fluid shunts in the BPD clinic population (*p* = 0.14).

### Other Clinical Features

Subjects with pulmonary hypertension were more likely to have a gastrostomy tube (*p*<0.001) or a Nissen fundoplication (*p*<0.001) than those without pulmonary hypertension in the BPD clinic population. However, in adjusted multivariable regression including both morbidities, only gastrostomy tubes (OR: 6.09; 95%CI: 3.71, 9.98; *p* = 0.002; n = 565) were associated with PH, while Nissen fundoplications were not (*p* = 0.83). Subjects in the NICU population with PH were more likely to be evaluated for sepsis (*p*<0.001) than their counterparts without PH, but not any more likely to have retinopathy of prematurity (*p* = 0.08) or necrotizing enterocolitis (*p* = 0.38). Each episode of sepsis was associated with 1.26-fold (95% CI: 1.11, 1.43; *p*<0.001; n = 229) increased likelihood of PH in adjusted regression.

### Risk Score for Pulmonary Hypertension

The BPD clinic population was used the training dataset since it was larger and enriched for pulmonary hypertension, and the NICU population as the validation dataset as it represents a more generalizable cohort of preterm infants. We derived a predictive algorithm by selecting 3 factors associated with PH in the BPD clinic population, namely birth weight, the use of home supplemental oxygen, and PDA ligation. Home supplemental oxygen was chosen as a measure of respiratory support as it was also ascertained in the NICU population. We combined these factors to form an additive risk factor score (range: 0–3) with 1 point assigned for birth weight ≤780 grams, 1 point for discharge on home supplemental oxygen, and 1 point for PDA ligation. The birth weight cutoff was selected based on an ROC curve of PH and birth weight in the BPD clinic population to maximize both sensitivity and specificity. The areas under the ROC curve for the BPD population and NICU population using the study score to predict PH were 0.738 (n = 562) and 0.864 (n = 228), respectively. Subjects with study scores of 2 or 3 were more likely to have PH than those with scores of 0 or 1 (**Table 3**; *p* value <0.001 for both populations).

**Table 3:** Pulmonary Hypertension and Study Score.

Study Score =Birth Weight (1 for ≤780 grams; 0 for >780 grams) +PDA Ligation (1 for Yes; 0 for No) +Home Supplemental Oxygen (1 for Yes; 1 for No)	(% of Subjects with Pulmonary Hypertension)	
	BPD Clinic Population(n = 562)	NICU Population(n = 228)
Study Score = 0	4.6%(n = 217)	1.0%(n = 101)
Study Score = 1	12.9%(n = 178)	1.5%(n = 68)
Study Score = 2	27.9%(n = 129)	25.0%(n = 44)
Study Score = 3	44.7%(n = 38)	40.0%(n = 15)

## Discussion

Pulmonary hypertension is a recognized source of morbidity and mortality among preterm infants, particularly among infants with bronchopulmonary dysplasia. However, there is not widespread consensus among healthcare providers with regard to screening for PH in preterm infants. Although PH only affects a minority of preterm infants overall, identifying those infants at highest risk for PH by using a targeted approach may improve overall outcomes through earlier diagnosis and treatment. In our study we observed a low PH prevalence of 8.3% in infants born with a birth weight less than 1000 grams, and 14.8% among preterm infants with BPD. Significant morbidity was also observed with increased initial hospitalization durations in infants with PH, both in the NICU population (1.7 months; *p*<0.001) and in the BPD clinic population (2.9 months; *p*<0.001). Thus, identifying risk factors for PH may be important in diagnosing and treating disease earlier.

We also found that PH was 3 times more likely to be observed in infants with a history of patent ductus arteriosus requiring ligation in both the NICU population (OR: 3.19) and the BPD clinic population (OR: 2.67). The persistence of a PDA in preterm infants may lead to an ongoing systemic-to-pulmonary shunt, which in turn could lead to increased pulmonary pressures and also has been associated, in one study with an increased risk of BPD.[27] The management of preterm infants with PDA and PH is not always straightforward. Case reports suggest that pulmonary hypertensive crises may result in right ventricular failure in patients with supra-systemic pulmonary pressures who have PDA ligation or pharmacological closure as a pulmonary-to-systemic shunt through a PDA could serve to relieve pulmonary pressures.[28,29] Additionally, the use of supplemental oxygen for the management of PH and BPD may have competing effects with respect to pulmonary blood flow as increased arterial oxygen saturation may lead to ductal constriction reducing blood flow, but also pulmonary vasodilation increasing blood flow.[30] It is unknown whether any of the pharmacological agents used for the management of PH (e.g., sildenafil, bosentan, etc.) have any effects on a PDA. Alternatively, it is possible that no relationship exists between PH and PDA ligation, despite being previously reported (unadjusted OR: 1.9; n = 114),[4] and seen in both populations in this study. Also, our definition of a PDA is based on infants receiving surgical ligation, which does not include patients with hemodynamically significant lesions who responded to pharmacological closure.

Corroborating results from other studies,[5,8,10,19,20] we also observed associations between lower birth weights and PH in both studied populations, thus highlighting the relevance of overall fetal growth to the development of lung vasculature as we did not identify any other demographic or socioeconomic factors to be associated with PH. Although markers of more severe lung disease (i.e., home ventilator or supplemental oxygen use, diuretic use) were associated with PH in the BPD clinic population, again similar to previous published work,[9,19,23] we did not observe similar factors (i.e., home supplemental oxygen use, the development of BPD) to be associated with PH in the NICU population, which may suggest that the association between severity of lung disease and PH is more easily seen in a population enriched for lung disease like the BPD clinic population. Although we did observe an association between gastrostomy tubes and PH, it is unclear whether there is a link between them such as aspiration or they are unrelated markers of more severe disease.

Combining our pertinent associations into a risk score using the BPD clinic population as a training dataset, we found within the NICU validation dataset that the risk score identified infants at highest risk for PH with 25–40% of subjects with higher scores being associated with PH, but only 1–1.5% of subjects with lower scores being associated with PH. It should be noted that while birth weight is assessed presumably before the development of PH, ascertaining the presence of PDA ligation and home supplemental oxygen may occur after the development of PH. However, perhaps difficulty in weaning FiO2 in the NICU setting or the persistence of a PDA despite medical management should be considered as elements to be considered in clinical decisions for who to screen for PH.

This study was limited by using already recruited study populations, although for the NICU population, data had been prospectively collected. Also these populations were recruited with different eligibility criteria, but the reproducibility of key findings across both populations may highlight the generalizability of our findings. Using solely clinically collected echocardiograms to determine the presence or absence of PH is another potential limitation of this study. First, while cardiac catheterization can more accurately determine PH severity, echocardiography has been demonstrated to qualitatively diagnose PH in infants with BPD.[31] Second, the use of clinically collected echocardiograms may underestimate the prevalence of PH, particularly that of mild PH, if echocardiograms were not obtained at appropriate intervals. Third, the timing of the diagnosis of PH was different in the 2 groups, but this may highlight the potential risk factors that were identified even though the echocardiograms may have been obtained at different times. Additionally, all subjects were recruited through tertiary care facilities, and these patients may have more severe disease than the population of preterm infants as a whole given referral patterns.

Overall, our results support previous findings that lower birth weights and more severe BPD are associated with pulmonary hypertension that persists outside of the neonatal period. This pulmonary hypertension is also associated with increased morbidity as measured by longer durations of initial hospitalizations after birth. We also observed an association between pulmonary hypertension and PDA requiring surgical ligation, but due to the nature of data collection a causal relationship cannot be established. We found that infants with 2 or more risk factors (low birth weight [≤780 grams], home supplemental oxygen use, and PDA ligation) were more likely to have pulmonary hypertension suggesting that infants with a combination of a risk factors may need to be more aggressively screened for pulmonary hypertension. Ultimately, additional prospective studies are necessary to determine whether earlier and/or more aggressive management of PDA and screening for pulmonary hypertension would improve outcomes with regard to pulmonary hypertension in preterm infants.

## Supporting Information

S1 Table Comparison of the NICU and BPD Clinic Study Populations. (DOCX)
