# Supplementary material for: Risk Factors and Clinical Outcomes in Preterm Infants with Pulmonary Hypertension
Source: PLoS One. 2016 Oct 7;11(10):e0163904. doi: 10.1371/journal.pone.0163904 (PMC5055317; doi:10.1371/journal.pone.0163904)
Supplement: S1 Table — (DOCX) [file pone.0163904.s001.docx]

**Supplemental Table 1.** Comparison of Populations

| Mean ± S.D.  [Range] | Entire NICU Population  (n = 230) | Entire BPD Clinic Population  (n = 580) | *P* Value |
| --- | --- | --- | --- |
| Sex (% female) | 50.9% | 39.1% | 0.002 |
| Gestational Age (weeks) | 25.9 ± 1.6  [22.0, 29.0] | 27.1 ± 2.9  [22.7, 36.9] | <0.001 |
| Birth Weight (grams) | 788 ± 145  [330, 1000] | 994 ± 511  [380, 4063]  (n = 565) | <0.001 |
| Birth Weight Percentile (%) | 41 ± 21  [1, 91] | 41 ± 23  [1, 96]  (n = 565) | 0.87 |
| Discharge Age (months) | 3.6 ± 1.5  [1.2, 12.1]  (n = 229) | 4.2 ± 2.9  [0.1, 24.5]  (n = 577) | 0.009 |
| Patent Ductus Arteriosus Requiring Ligation (% yes) | 22.7%  (n = 229) | 19.6%  (n = 576) | 0.33 |
| Bronchopulmonary Dysplasia (% yes) | 67.4% | 100% | <0.001 |
| Home Supplemental Oxygen (% yes) | 23.1%  (n = 229) | 35.7% | 0.001 |
| Tracheostomy (% yes) | 3.5% | 4.3% | 0.59 |
| Pulmonary Hypertension (% yes) | 8.3% | 14.8% | 0.012 |
